# Impact of varying diastolic pressure fitting technique for the reservoir-wave model on wave intensity analysis

**DOI:** 10.1177/0954411920959957

**Published:** 2020-09-30

**Authors:** Nicola Pomella, Ernst R Rietzschel, Patrick Segers, Ashraf William Khir

**Affiliations:** 1 Biomedical Engineering Research Group, Brunel University London, UK; 2 Centre for Genomics and Child Health, Blizard Institute, Queen Mary University of London, UK; 3 Department of Cardiovascular Diseases, Ghent University Hospital, Ghent, Belgium; 4 IBiTech-bioMMeda, Ghent University, Ghent, Belgium; * Current affiliation: Centre for Genomics and Child Health, Blizard Institute, Queen Mary University of London, UK

**Keywords:** Blood flow measurement, cardiovascular system mechanics, hemodynamics, mathematical modeling [medical], medical signal processing, reservoir pressure, asymptotic pressure, wave intensity analysis

## Abstract

The reservoir-wave model assumes that the measured arterial pressure is made of two components: reservoir and excess. The effect of the reservoir volume should be excluded to quantify the effects of forward and backward traveling waves on blood pressure. Whilst the validity of the reservoir-wave concept is still debated, there is no consensus on the best fitting method for the calculation of the reservoir pressure waveform. Therefore, the aim of this parametric study is to examine the effects of varying the fitting technique on the calculation of reservoir and excess components of pressure and velocity waveforms. Common carotid pressure and flow velocity were measured using applanation tonometry and doppler ultrasound, respectively, in 1037 healthy humans collected randomly from the Asklepios population, aged 35 to 55 years old. Different fitting techniques to the diastolic decay of the measured arterial pressure were used to determine the asymptotic pressure decay, which in turn was used to determine the reservoir pressure waveform. The corresponding wave speed was determined using the PU-loop method, and wave intensity parameters were calculated and compared. Different fitting methods resulted in significant changes in the shape of the reservoir pressure waveform; however, its peak and time integral remained constant in this study. Although peak and integral of excess pressure, velocity components and wave intensity changed significantly with changing the diastolic decay fitting method, wave speed was not substantially modified. We conclude that wave speed, peak reservoir pressure and its time integral are independent of the diastolic pressure decay fitting techniques examined in this study. Therefore, these parameters are considered more reliable diagnostic indicators than excess pressure and velocity which are more sensitive to fitting techniques.

## Introduction

Arterial blood pressure waveform, which is affected by many physiological and pathological factors, changes its morphology as it travels along the arterial tree. The mechanical properties of the vessels contribute to these changes; for example, the arterial elasticity reduces the pressure pulsation along the systemic tree. Waves originated by the contracting heart travel forward toward the peripheral arteries, reflect at sites of mismatched impedance (where the arteries, for example, bifurcate or taper) and travel backward toward the heart. The interaction between the forward- and backward traveling waves further induce changes to the magnitude and shape of the pressure waveform. Modeling the pressure waveform is therefore a complex task and different models have been proposed,^1^ such as 0D (Windkessel), 1D, and 3D.

In the context of analyzing the pressure waveform, the reservoir-wave approach assumes that the measured arterial pressure can be divided into two components: the reservoir pressure ($P_{r}$), affected by the elastic properties of the vessel, hence dependent on the arterial reservoir volume, and the excess pressure ($P_{\mathrm{ex}}$), thought of being the product of the traveling waves^2,3^; therefore, the analysis of the effects of forward- or backward-traveling waves on arterial pressure (and blood flow velocity) is carried out by excluding $P_{r}$.

The reservoir-wave model was first applied in canine aorta,^2^ considering the reasonable assumption that the blood flow is null in diastole. Consequently, it was applied for the calculation of venous reservoir^4^ and to any arbitrary arterial location.^5,6^ However, the calculation of $P_{r}$ requires fitting the diastolic decay of the measured pressure waveform to calculate the parameters $P_{\infty }$ (asymptotical value) and b (rate constant) and there is no consensus over the value of these parameters. Some researchers didn’t fit the diastolic decay for the determination of $P_{\infty }$ but kept it fixed.

Specifically, Wang et al.^2^ fitted the diastolic decay over the last two thirds of the measured pressure, considering that waves are minimal during this period. The parameters R, C, and $P_{\infty }$, namely, the peripheral systemic resistance, the arterial tree compliance and the asymptotic value, were free-fitted. The value of the reservoir pressure at the onset of diastole (${\bar{P}}_{n}=P_{r}\left(T_{N}\right),$ where $T_{N}$ is the dicrotic notch) was fixed at the value of the measured pressure at the same time point ($P_{n}$). The same approach was used by Wang et al.^4,7^ for both venous and arterial reservoir pressure in the canine cardiovascular system, although the fitting window was reduced to approximately the last third of diastole. Davies et al.^8^ followed the approach used in Wang et al.,^2^ whereas Bia et al.^9^ the later technique of Wang et al.^7^

It is hypothesized that varying the fitting method would significantly change $P_{\infty }$ and b values, leading to different reservoir and excess pressure waveforms, as well as to different hemodynamic and wave intensity parameters, derived from the separated waveforms. Therefore, the aim of this study was to examine the effects of varying fitting technique on the calculation of reservoir and excess components of blood pressure and flow velocity, measured at the common carotid artery of healthy humans. The separated components were subsequently used to determine relevant hemodynamic and wave intensity parameters. The variation of the fitting method was based on the change of the number of its free-fitted parameters (called degrees of freedom, DOF, for brevity) −$P_{\infty }$, b, ${\bar{P}}_{n}$− and of the length of the fitting window (whole diastole or its final two thirds).

## Materials and methods

### Study group

The Asklepios Study is a longitudinal population study focusing on the interaction between ageing, cardiovascular hemodynamics, and inflammation in preclinical cardiovascular disease.^10^ A subset comprising 1037 subjects of the total cohort (2524 participants, 1301 women, spanning 4 half-decades: 35–55 years-old) provided data for this study.

Subjects of the Asklepios population were free from manifest cardiovascular disease at study initiation, randomly sampled from the twinned Belgian communities of Erpe–Mere and Nieuwerkerken. All examinations were single-observer, single-device, single-site, and were performed in a single 2-year consecutive timeframe.^10^ The procedure included measurements of basic clinical data, blood samples examination, echocardiographic examination, vascular echographic, and tonometric measurements. The study protocol was approved by the ethics committee of Ghent University Hospital and all subjects gave a written informed consent.

Table 1 summarizes the physiological and hemodynamic characteristics of the study group.

**Table 1.** Basic characteristics of the study group (*n* = 1037).

		*n*	Age (years)	Height (cm)	Weight (kg)	SBP (mmHg)	DBP (mmHg)	MAP (mmHg)	HR (bpm)
1st HD	T	311	38±2	171 ± 9	73 ± 13	127±13	74±10	96±10	63 ± 9
	F	162	38±2	165 ± 6	66 ± 10	123±12	74±10	95±11	65±10
	M	149	38±2	178 ± 6	81 ± 11	132±12	75±10	98 ± 10	61±8
2nd HD	T	262	44±2	170±9	72±13	129±14	77±10	99±11	64 ±11
	F	142	43±2	165±6	65±10	126±15	75±10	97±12	66±10
	M	120	44±1	176±7	81 ± 11	133±12	79±10	101±11	61±12
3rd HD	T	254	48 ± 1	170 ± 9	75 ± 14	131 ± 13	77 ± 10	100 ±10	65±9
	F	120	48 ± 1	163 ± 6	66 ± 11	128 ± 13	75 ± 9	98± 10	66±8
	M	134	48 ± 1	175 ± 6	82 ± 11	135 ± 13	79 ± 10	102± 11	64±11
4th HD	T	210	54±2	168 ± 9	73 ± 13	136 ± 16	79 ± 10	104± 12	64±10
	F	107	53±2	161 ± 6	66 ± 10	136 ± 18	78 ± 11	104±14	65±8
	M	103	54±2	175 ± 6	81±11	137 ± 14	80 ± 9	104±10	62±12

DBP: brachial diastolic blood pressure; F: female; HD: half-decade; HR: heart rate; M: male; MAP: brachial mean blood pressure; SBP: brachial systolic blood pressure; T: total. Values are reported as mean ±SD

### Hemodynamic measurements

Details of the protocol can be found in Rietzschel et al.^10^ Briefly, blood pressure and flow velocity measurements were acquired via applanation tonometry and vascular echography, respectively. The measurements were not simultaneously taken, but acquired during the same vascular examination. The signals were post-processed and subsequently aligned using the algorithm proposed by Swalen and Khir.^11^ The tonometric procedure, carried out with a Millar pentype tonometer (SPT 301, Millar Instruments, Houston, Texas, USA), consisted of the following two steps^12^: (1) tracings were collected from the brachial artery for 20 s, at a sampling rate of 200 Hz, then divided into individual beats, using the foot of the wave as fiducial marker, and ensemble-averaged. The averaged tracing was calibrated against oscillometrically measured brachial systolic and diastolic (DBP_b_) pressure and mean arterial brachial pressure (MAP_b_) was calculated by numerically averaging the curve; and (2) tonometry was performed on the carotid artery as described in the previous step and tracings were ensemble-averaged and calibrated against the previously calculated tonometric brachial pressure, assuming that diastolic and mean pressure values are fairly constant in large arteries. A scaled carotid pressure waveform (P) was finally obtained.

A commercially available ultrasound system (VIVID 7, GE Vingmed Ultrasound, Horten, Norway), equipped with a linear vascular transducer (12 L, 10 MHz), was used for the scans. Blood flow velocity was measured via Pulsed Wave Doppler with sweep speed equal to 100 mm/s and 5 to 30 ECG-gated cardiac cycles were recorded during normal breathing. The DICOM images were subsequently processed^13^ with custom written programs in Matlab (The MathWorks, Natick, Massachusetts, USA). The velocity profile was obtained by averaging the maximum and minimum velocity envelopes and it was finally divided into individual cardiac cycles that were successively ensemble-averaged to obtain a single velocity contour (U).

### Data analysis

Data analysis was performed via custom-made algorithms in Matlab. The algorithm decomposed P and U waveforms into their reservoir and excess components. The reservoir pressure was calculated following^5^:

(1) $$\begin{array}{c} P_{r}=\frac{b}{a+b}P_{\infty }+e^{-\left(a+b\right)t}\\ \left[\int_{0}^{t}\mathrm{aP}\left(\vartheta \right)e^{\left(a+b\right)\vartheta }d\vartheta +P_{0}-\frac{b}{a+b}P_{\infty }\right] \end{array}$$

where a,b and $P_{\infty }$ are the systolic rate constant [s^−1^], the diastolic rate constant [s^−1^] and the asymptotic pressure value, respectively. In diastole (for $T_{N}<t<T$, where $T_{N}$ and T are the dicrotic notch time point and the duration of cardiac cycle, respectively), equation (1) reads:

(2) $$P_{r}=\left({\bar{P}}_{n}-P_{\infty }\right)e^{-b\left(t-T_{N}\right)}+P_{\infty }$$

where ${\bar{P}}_{n}$ is the reservoir pressure value at $t=T_{N}$, a time point defined as the local minimum in the measured pressure around the dicrotic notch.

Subsequently, the parameter a was calculated through the following equation:

(3) $$\begin{array}{c} P_{r}\left(T_{N}\right)=\frac{b}{a+b}P_{\infty }+e^{-\left(a+b\right)T_{N}}\\ \;\left[\int_{0}^{T_{N}}\mathrm{aP}\left(\vartheta \right)e^{\left(a+b\right)\vartheta }d\vartheta +P_{0}-\frac{b}{a+b}P_{\infty }\right] \end{array}$$

The complete reservoir waveform could be obtained via equation (1) and the excess pressure was determined as the difference between the measured pressure P and the reservoir pressure: $P_{\mathrm{ex}}=P-P_{r}$. Similar analysis held for the velocity components $U_{r}$ and $U_{\mathrm{ex}}$, where $U=U_{r}+U_{\mathrm{ex}}$ and $U_{r}=\left(P-P_{\infty }\right)/\bar{R}$, where $\bar{R}$ is the averaged downstream resistance, calculated as $\left(〈P〉-P_{\infty }\right)/〈U〉$, where 〈P〉 and 〈U〉 are the time-averaged measured pressure and velocity, respectively, during diastole. A second approach to calculate $\bar{R}$, involving the determination of the linear part of the PU-loop in diastole and proposed by the same investigators, was not used in this study because it was difficult to assess the linear part of the loop in diastole.

### Fitting algorithm settings

The fitting parameters b, ${\bar{P}}_{n}$, and $P_{\infty }$ were determined, for each subject, using the following fitting procedure (details are given in Table 2): the value of ${\bar{P}}_{n}$ was either determined by fitting (3 DOF analysis) or fixed (2 DOF analysis), whereas the length of the fitting window was either set equal to the entire diastolic window (“whole” diastole) or to its last two thirds (2/3 diastole, abbreviated as “23”). The combination of these settings gave four different fitting methods: 3 DOF whole, 3 DOF 23, 2 DOF whole, 2 DOF 23. In the text, the abbreviation “whole” is left out for brevity; therefore, the denominations “3 DOF” and “2 DOF”– without the label “23”– imply the usage of the “whole” diastolic window.

**Table 2.** Properties of the analyses performed.

Analysis	Free-fitted parameters	Fixed paramaters	Window length
3 DOF	${\bar{P}}_{n}$, *P_∞_, b*	–	Whole diastole
2 DOF	*P_∞_, b*	${\bar{P}}_{n}=P_{n}$	Whole diastole
3 DOF 23	${\bar{P}}_{n}$, *P_∞_, b*	–	Last 2/3 of diastole
2 DOF 23	*P_∞_, b*	${\bar{P}}_{n}=P_{n}$	Last 2/3 of diastole

*b*: diastolic rate constant; *P_∞_*: asymptotical pressure value; *${\bar{P}}_{n}$*: reservoir pressure value at the dicrotic notch.

**Figure 1.**
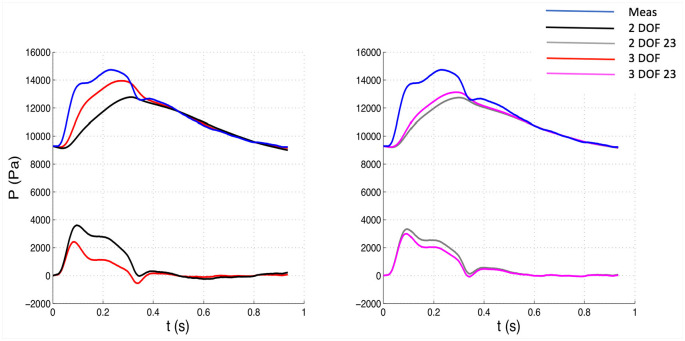
Comparison of pressure waveforms between 3 DOF and 2 DOF settings, whole window (Left) and between 3 DOF and 2 DOF, 23 window (Right) for one patient. The measured pressure is depicted in blue. The top waveforms depicted along with the measured pressure represent the reservoir components, whereas the bottom waveforms the excess components. Meas: measured waveform.

**Figure 2.**
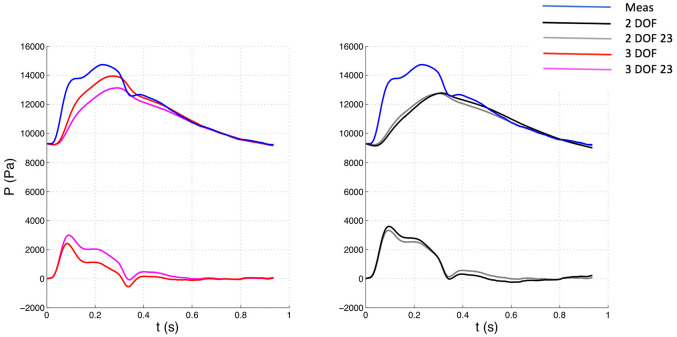
Comparison of pressure waveforms between whole and 23 window with 3 DOF (Left) and 2 DOF (Right) settings for one patient. The measured pressure is depicted in blue. The top waveforms depicted along with the measured pressure represent the reservoir components, whereas the bottom waveforms the excess components. Meas: measured waveform.

**Figure 3.**
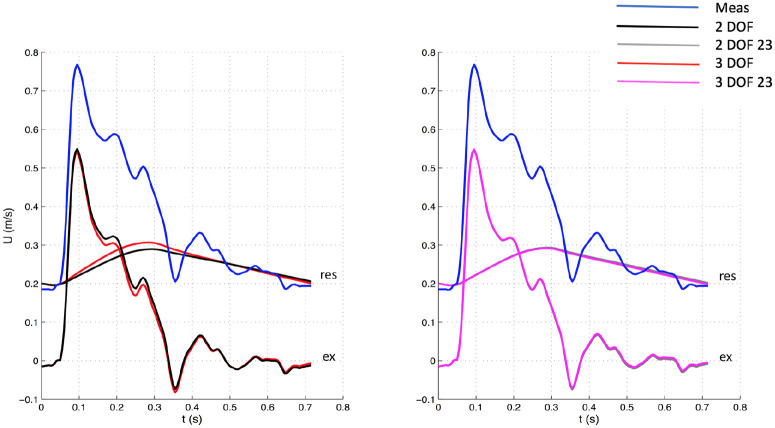
Comparison of velocity waveforms between 3 DOF and 2 DOF settings, whole window (Left) and between 3 DOF and 2 DOF, 23 window (Right) for one patient. The measured velocity is depicted in blue. The top waveforms (“res”) depicted along with the measured velocity represent the reservoir components, whereas the bottom waveforms (“ex”) the excess components. Meas: measured waveform.

**Figure 4.**
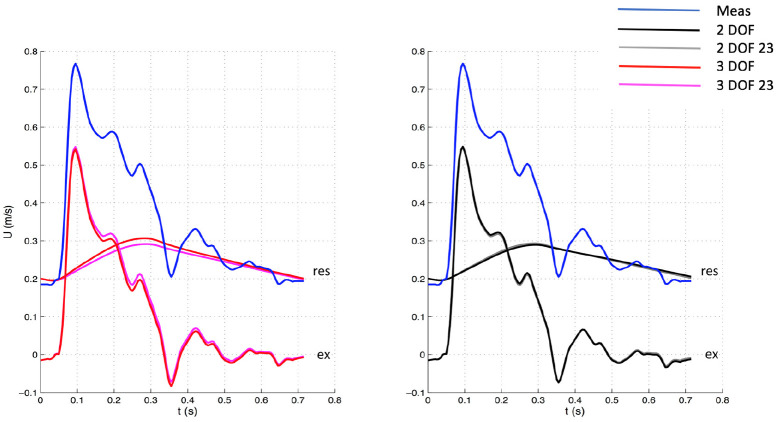
Comparison of velocity waveforms between whole and 23 window with 3 DOF (Left) and 2 DOF (Right) settings for one patient. The measured velocity is depicted in blue. The top waveforms (“res”) depicted along with the measured velocity represent the reservoir components, whereas the bottom waveforms (“ex”) the excess components. Meas: measured waveform.

The 3 DOF and 3 DOF 23 analyses were characterized by 3 degrees of freedom, because b, ${\bar{P}}_{n}$, and $P_{\infty }$ were determined by fitting, whereas in the 2 DOF and 2 DOF 23 analyses only b and $P_{\infty }$ were calculated with the fitting algorithm while ${\bar{P}}_{n}$ was set equal to the corresponding value of measured pressure $P_{n}.$ See Figures 1–4 for examples.

The fitting algorithms were implemented using the *lsqcurvefit* function, a Matlab solver optimized for non-linear least squares problems. $P_{\infty }$ and b were bound to be non-negative and the initial conditions for the solver were the following: $P_{\infty }$ = 60 mmHg (7999.2 Pa) and b = 1 s^−1^. Where ${\bar{P}}_{n}$ was free-fitted, it was also bound to be non-negative and the initial condition was ${\bar{P}}_{n}$ = $P_{n}$. For the determination of the systolic rate constant a, the function *lsqcurvefit* was used to solve equation. 3, with initial condition a = 10 s^−1^.^5^ Relative tolerance was set at 10^−12^.

The following hemodynamic parameters were calculated: the maxima of $P_{r}$ and $P_{\mathrm{ex}}$ ($P_{r}\max$ and $P_{\mathrm{ex}}\max$, respectively), the integral with respect to time of reservoir and excess pressure curves (PRI and PEI, respectively), the maxima of $U_{r}$ and $U_{\mathrm{ex}}$ ($U_{r}\max$ and $U_{\mathrm{ex}}\max$, respectively) and the index $X^{2}$, the mean square error between measured and reservoir pressure in diastole, according to the equation:

(4) $$X^{2}=\frac{1}{N}\sum_{i=1}^{N}{\left(P_{i}-{P_{r}}_{i}\right)}^{2}$$

Where N is the number of data points in diastole.

### Wave intensity analysis

Wave intensity analysis was performed on both measured waveforms (P,U) and calculated excess waveforms ($P_{\mathrm{ex}},U_{\mathrm{ex}})$ for each subject.

**Figure 5.**
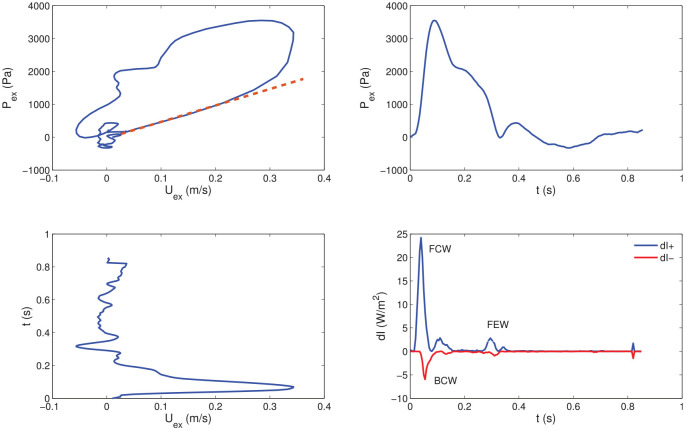
Example of P_ex_U_ex_ loop (Top Left), P_ex_ contour (Top Right), U_ex_ contour (Bottom Left) and corresponding wave intensity (Bottom Right; obtained via equation (6)) for one patient. A straight line highlighting the slope of the linear portion is superimposed on the P_ex_U_ex_ loop (see equation (5)). Forward compression (FCW), backward compression (BCW) and forward expansion (FEW) waves are labelled in the wave intensity plot. dI+: forward wave intensity component, dI−: backward wave intensity component.

Assuming that only forward-traveling waves are present during the early systolic portion of each cardiac cycle,^14^ the slopes of the linear part of the PU- and P_ex_U_ex_-loops were used to calculate the corresponding wave speed values [m/s] using the following equation:

(5) $$c=\frac{1}{\rho }\frac{\Delta P_{+}}{\Delta U_{+}}$$

over the early systolic part of the loops (Figure 5). The numerator and denominator in equation (5) refer to the time of the cardiac cycle when waves are running only in the forward direction and the relationship between the measured P and U waveforms is linear for the PU-loop and for the excess waveforms of P_ex_U_ex_-loop. Blood density ρ was set equal to 1050 kg/m^3^. Subsequently, the wave intensity dI=dPdU [W/m^2^] was separated into its forward-traveling and backward-traveling components^15^:

(6) $$dI_{\pm }=dP_{\pm }dU_{\pm }=\pm \frac{1}{4\rho c}{\left(\mathrm{dP}\pm \rho \mathrm{cdU}\right)}^{2}$$

After the calculation of wave speed and wave intensity, relevant wave intensity parameters were extracted. The area of the forward compression wave (FCW), which is generated by the contraction of the left ventricle, was derived by integrating the early-systolic peak observed in $dI_{+}$ over time (Figure 5). Therefore, it has units of [$W\;\cdot \;s\;\cdot \;m^{-2}=J\;\cdot \;m^{-2}$ ]. Similarly, the area of the backward compression wave (BCW), attributed to reflections from the head microcirculation, was determined by integrating over time the mid-systolic peak present in $dI_{-}$. Finally, the area of the forward expansion wave (FEW), correlated to the decrease in shortening velocity of the left ventricle in late systole, was determined by integrating the late-systolic peak seen in $dI_{+}$ over time.

### Statistical analysis

All values are reported as mean ± SD, relative to the whole cohort, in the text, tables, and figures. The statistical analysis was performed using SPSS Statistics (version 20, IBM, Armonk, New York, USA). Hemodynamic and wave intensity parameters were statistically compared via one-way analysis of variance (ANOVA) and Tukey’s post-hoc test. A paired two-tailed *t*-test was also performed for the comparison between PU-derived- and P_ex_U_ex_-derived parameters. Statistical significance was assumed if adjusted *p*-value<0.05.

## Results

**Figure 6.**
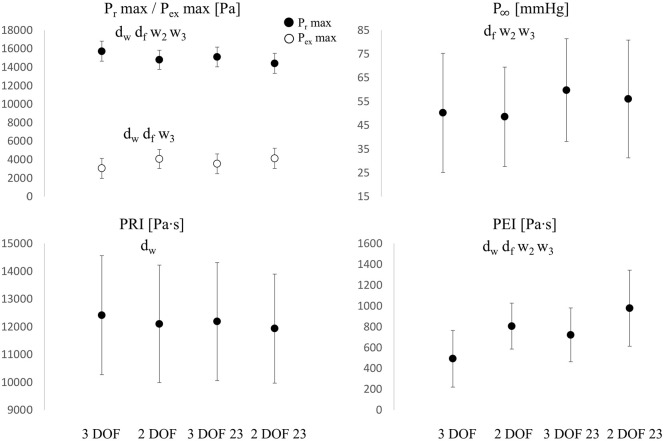
Comparisons of $P_{r}\max$ and $P_{\mathrm{ex}}\max$ (Top Left), $P_{\infty }$ (Top Right), PRI (Bottom Left) and PEI (Bottom Right) values among all fitting settings. d_w_: significant difference between 3 DOF and 2 DOF, whole window (*p*  < 0.05), d_f_: significant difference between 3 DOF and 2 DOF in 23 window, w_2_: significant difference between 2 DOF and 2 DOF 23, w_3_: significant difference between 3 DOF and 3 DOF 23. Y-axis units are reported in each figure title. Values are reported as mean ± SD, indicated by the error bars.

$P_{\infty }$ (Figure 6) slightly decreased from 3 DOF to 2 DOF in both window settings (whole and 23), non-significantly (*p*  > 0.05) in the former case (−4%) and significantly (*p*  < 0.05) in the latter case (−6%), whereas the change from whole to 23 diastolic window caused bigger variations, specifically +19% (*p*<0.05) and +15% (*p*  < 0.05) for 3 DOF and 2 DOF, respectively.

**Figure 7.**
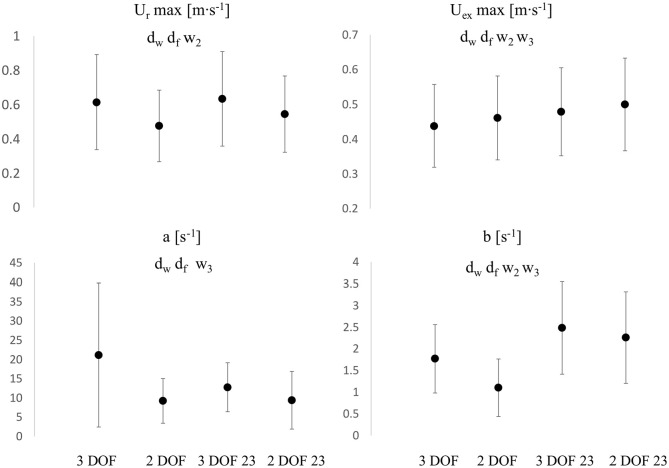
Comparisons of $U_{r}\max$ (Top Left), $U_{\mathrm{ex}}\max$ (Top Right), a(Bottom Left) and b(Bottom Right) values among all fitting settings. d_w_: significant difference between 3 DOF and 2 DOF, whole window (*p*  < 0.05), d_f_: significant difference between 3 DOF and 2 DOF in 23 window, w_2_: significant difference between 2 DOF and 2 DOF 23, w_3_: significant difference between 3 DOF and 3 DOF 23. Y-axis units are reported in each figure title. Values are reported as mean ± SD, indicated by the error bars.

The diastolic constant b (Figure 7) significantly decreased from 3 DOF to 2 DOF in both window settings: −38% for the whole window and −9% for 23. The change in window (from whole to 23) caused bigger variations: +40% (*p*  < 0.05) and +105% (*p* < 0.05) for 3 DOF and 2 DOF, respectively. Therefore, b seemed affected by both changes in DOF and window settings, whereas $P_{\infty }$ substantially changed only with changes in window. The systolic constant *a* (Figure 7) significantly decreased from 3 DOF to 2 DOF in both window settings: −57% for the whole window and −26% for 23. The changes in window caused a variation of −40% for 3 DOF (*p*  < 0.05) whereas it was not significant for 2 DOF.

$P_{r}\max$ (Figure 6) significantly decreased from 3 DOF to 2 DOF, in both window settings: −6% for the whole window and −5% for 23. The variation exhibited from whole to 23 was −4% (*p*  < 0.05) for 3 DOF and −3% (*p*  < 0.05) for 2 DOF. $U_{r}\max$ (Figure 7) showed a slightly different pattern: a variation of −22% from 3 DOF to 2 DOF with the whole window and −14% with a 23 window, while the variation exhibited from whole to 23 window was +3% (*p*  > 0.05) for 3 DOF and +14% (*p*  < 0.05) for 2 DOF.

$P_{\mathrm{ex}}\max$ (Figure 6) and $U_{\mathrm{ex}}\max$ (Figure 7) significantly increased from 3 DOF to 2 DOF, in both window settings: +32% and +5% with the whole window, and +16% and +4% with 23 window, for $P_{\mathrm{ex}}\max$ and $U_{\mathrm{ex}}\max$, respectively. The variations exhibited from whole to 23 window were: +16% (*p*  < 0.05) and +9% (*p*  < 0.05) for 3 DOF, +1% (*p*  > 0.05) and +8% (*p*  < 0.05) for 2 DOF, for $P_{\mathrm{ex}}\max$ and $U_{\mathrm{ex}}\max$, respectively. Therefore, $P_{\mathrm{ex}}\max$ showed overall a bigger variation than $U_{\mathrm{ex}}\max$.

PEI (Figure 6) significantly increased from 3 DOF to 2 DOF, in both window settings: +64% for the whole window and +36% for 23 window. The change exhibited from the whole to 23 window was +46% (*p*  < 0.05) and +21% (*p*  > 0.05) for 3 DOF and 2 DOF, respectively. The variation of PRI (Figure 6) was much less pronounced: it was −3% (*p*  < 0.05) and −2% (*p*  > 0.05) from 3 DOF to 2 DOF, for the whole window and 23 window, respectively, whereas the variation exhibited from the whole to 23 window was not significant: −2% (*p*  > 0.05) and −1% (*p*  > 0.05) for 3 DOF and 2 DOF, respectively.

**Figure 8.**
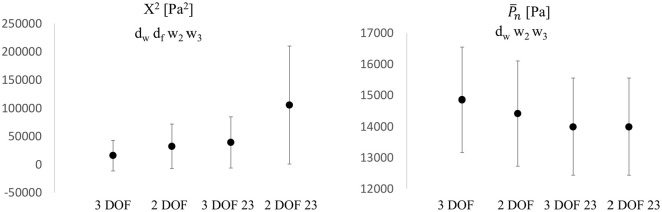
Comparisons of $X^{2}$ (Left) and ${\bar{P}}_{n}$(Right) values among all fitting settings. d_w_: significant difference between 3 DOF and 2 DOF, whole window (*p*  < 0.05), d_f_: significant difference between 3 DOF and 2 DOF in 23 window, w_2_: significant difference between 2 DOF and 2 DOF 23, w_3_: significant difference between 3 DOF and 3 DOF 23. Y-axis units are reported in each figure title. Values are reported as mean ± SD, indicated by the error bars.

The error $X^{2}$ (Figure 8) increased from 3 DOF to 2 DOF in both window settings: +104% and +168% for the whole and 23 window, respectively (both *p*  < 0.05). Also, it increased from whole to 23 window in both DOF settings (+150% and +227% for 3 DOF and 2 DOF, respectively, *p*  < 0.05). Therefore, the lowest values for the index were found with 3 DOF-whole window settings.

${\bar{P}}_{n}$ (Figure 8) tended to significantly increase when free-fitted (+3%) compared with the fixed (measured) value $P_{n}$, bringing a substantial negative region in $P_{\mathrm{ex}}$. The change is not significant for a 23 window. On the contrary, ${\bar{P}}_{n}$ tended to significantly decrease when the window was shortened (−3%) for 3 DOF (being the value of dicrotic notch fixed in 2 DOF). Nevertheless, the variation in window setting (from whole to 23) brought a slight change in the shape of the reservoir waveform, even when the dicrotic notch was fixed.

**Figure 9.**
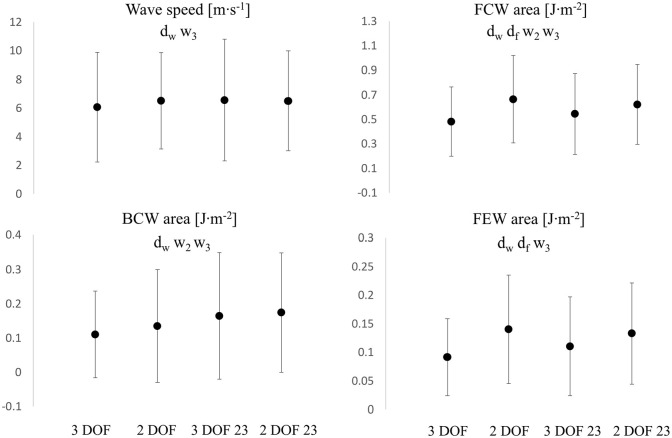
Comparisons of wave speed (Top Left), FCW area (Top Right), BCW area (Bottom Left) and FEW area (Bottom Right) values among all fitting settings. d_w_: significant difference between 3 DOF and 2 DOF, whole window (*p*  < 0.05), d_f_: significant difference between 3 DOF and 2 DOF in 23 window, w_2_: significant difference between 2 DOF and 2 DOF 23, w_3_: significant difference between 3 DOF and 3 DOF 23. Y-axis units are reported in each figure title. Values are reported as mean ± SD, indicated by the error bars.

In the context of wave speed and wave intensity analysis (calculated from P_ex_ and U_ex_), wave speed (Figure 9) significantly increased (+7%, *p*  < 0.05) from 3 DOF to 2 DOF for the whole window setting and remained fairly unchanged (−1%, *p*  > 0.05) with 23 window. Also, it increased (+8%, *p*  < 0.05) in 3 DOF and remained unchanged (−1%, *p*  > 0.05) in 2 DOF, from whole to 23 window. Overall, wave speed values did not substantially change in-between settings.

FCW area (Figure 9) significantly increased from 3 DOF to 2 DOF, in both window settings: +38% with the whole window and +14% with 23 window. The change exhibited from the whole to 23 window was +13% (*p*  < 0.05) for 3 DOF and −6% (*p*  < 0.05) for 2 DOF. A very similar pattern was recorded for the forward expansion wave. FEW area (Figure 9) significantly increased from 3 DOF to 2 DOF, in both window settings: +53% for the whole window and +20% for 23 window. The change exhibited from whole to 23 window was +21% (*p*  < 0.05) for 3 DOF and −5% (*p*  > 0.05) for 2 DOF. BCW area (Figure 9) increased from 3 DOF to 2 DOF in both window settings: +22% (*p*  < 0.05) and +6% (*p*  > 0.05) for whole and 23 window, respectively. The variation exhibited with change in window (from whole to 23) was significant: +49% (*p*  < 0.05) and +29% (*p*  < 0.05) for 3 DOF and 2 DOF, respectively.

PU-derived parameters were always greater than corresponding P_ex_U_ex_-derived parameters: the biggest difference was recorded with 3 DOF whole window setting (between +19.5% for wave speed and +89.5% for BCW area). Table 3 reports the percentage variations that PU-derived parameters exhibited with respect to corresponding P_ex_U_ex_-derived parameters.

**Table 3.** Comparison of PU-derived and P_ex_U_ex_-derived wave intensity parameters.

Fitting method	Wave speed	FCW area	FEW area	BCW area
3 DOF	+19.5	+73.4	+58.8	+89.5
2 DOF	+11.3	+25.8	+3.5^†^	+54.9
3 DOF 23	+10.5	+53.5	+31.5	+27.1
2 DOF 23	+11.4	+34.6	+9.1	+19.7

Values are reported as percentage (%) variations of parameters obtained with PU loop (measured waveforms), with respect to corresponding parameters obtained with excess components P_ex_ and U_ex_. ^†^: not significant (*p*  > 0.05). As indicated, only one variation was not significant (FEW area in 2 DOF setting).

## Discussion

This parametric study aimed to compare various common carotid hemodynamic and wave intensity parameters, using different fitting techniques for the calculation of the reservoir pressure and velocity waveforms, assuming the reservoir-wave hypothesis (with a single exponential decay) for the decomposition of the arterial pressure.

Whilst $P_{\mathrm{ex}}$ was thought to be formed due to the traveling waves, $P_{r}$ was initially thought to correspond to the Windkessel pressure,^2^ attributed solely to the elastic properties of the arteries. However, it was observed that $P_{r}$ traveled along the aorta,^16^ unlike the Windkessel model which assumes that the reservoir pressure varies only with time and is uniform spacially throughout the arterial system. Therefore, it is reasonable to infer that $P_{r}$ might also be affected by the forward and backward traveling waves; a concept that is evolving and yet to be established.

In general, the variation of fitting methods brought substantial modifications to the calculated waveforms, mainly highlighted by $X^{2}$ (equation (4)), because the differences between single data points of corresponding waveforms are squared.

The 23 window ensures an almost perfect match between measured pressure and reservoir waveform in the last two thirds of diastole, but leaves a bigger “gap” in the first diastolic third (Figure 1), compared to the whole window. The differences in gaps are more visible in Figure 2 (3 DOF 23 vs 3 DOF reservoir contour). The gap is associated with a slightly positive excess pressure waveform in the first diastolic third and may be related to the presence of little wave activity in the beginning of diastole.

The changes in the shape of both reservoir and excess waveforms affected hemodynamic and wave intensity parameters. Fixing the dicrotic notch (2 DOF setting, ${\bar{P}}_{n}=P_{n}$) generally brought a decrease in values of hemodynamic parameters such as $P_{r}\max,P_{\infty },b,\mathrm{PRI}$ and $U_{r}\max$. The variations were generally small (<10%), except for the rate constant b (up to 38%), whole window, and $U_{r}\max$ for both window settings (up to 22%). Shortening the window length (from the whole to the last two thirds of diastole) caused a substantial increment of the fitting parameters $\left(P_{\infty },b\right)$ up to 19% and 105%, respectively, as well as a modest increment for $U_{r}\max$. $P_{r}\max$ slightly decreased instead (up to 4%), whereas PRI did not significantly change. The variations caused by the change in window length were generally bigger than the variations occurred with a change in DOF.

Variations did not occur independently, as can be easily seen by re-arranging equation (2):

(7) $$b=-\frac{1}{\hat{t}-T_{N}}\ln\left(\frac{{\hat{P}}_{r}-P_{\infty }}{{\bar{P}}_{n}-P_{\infty }}\right)$$

where $\left({\hat{P}}_{r},\hat{t}\right)$ is a given data point of the reservoir pressure signal. Equation (7) is a positive monotonic function, because ${\hat{P}}_{r}<{\bar{P}}_{n}$ when $\hat{t}>T_{N}$; therefore, b increases for increasing $P_{\infty }$ values. The rate of increment is generally slow, except for the region in proximity of the vertical asymptote, given by the value of ${\bar{P}}_{n}$: when $P_{\infty }$ approaches this threshold, b becomes increasingly bigger.

The significant variations in hemodynamic parameters caused by changes in fitting techniques had effects on wave intensity, resulting in substantial differences in all main parameters. However, wave speed values did not substantially change with fitting methods, suggesting that it seems insensitive to those. As can be seen in Figures 1–4, excess pressure and velocity waveforms tended to preserve the slope of the upstroke, being also similar to that of corresponding measured waveforms. Table 3 shows that wave speed, being measured with both PU loop and $P_{\mathrm{ex}}U_{\mathrm{ex}}$ loop, did not change substantially, compared to wave intensity parameters. Generally, parameters calculated with the (measured) PU loop exhibited greater values than corresponding variables calculated with excess ($P_{\mathrm{ex}}$ and $U_{\mathrm{ex}}$) components. This is in agreement with Borlotti et al.,^17^ who performed wave intensity analysis using excess pressure components in the canine aorta, but did not perform the wave separation of U. As stated by the same investigators, the wave speed should not change when measured in a specific vessel using different techniques, as it represents a direct measure of arterial stiffness. However, it is important to consider that the excess components are able to better satisfy the assumption behind the loop technique, that only forward waves should be present. Also, because of reservoir pressure being related to systemic properties and its changes being the result of a combination of systemic variations, Bia et al.^9^ stated that the excess pressure is more useful for assessing local responses. Although the use of excess velocity waveform is still very limited in literature, we think it should be considered for local wave intensity analysis, because the reservoir component may be affected by reflected and re-reflected wavelets, masking the local effects.

The age of patients used for this study spans across two decades and the data show a broad spectrum of basic characteristics (Table 1). Therefore, it is important to higlight the wide variation in each of the parameters under study across the cohort. A couple of selected extreme cases has been reported in Table 4 to demonstrate the range of the results. Further, although another window size is used for the fitting of the reservoir pressure waveform in the literature, namely the last third (1/3) of diastole (as specified in the “Introduction”), it was not considered in this work. It was believed that a further comparison, between 2/3 and 1/3 of diastole, was not necessary at this stage and the difference was not expected to be significant to alter the current data interpretation or conclusions.

**Table 4.** Range (variability) demonstration: Two extreme cases of the cohort.

	Case 1 (F; 4th half-decade)				Case 2 (F; 3rd half-decade)			
	3 DOF	2 DOF	3 DOF 23	2 DOF 23	3 DOF	2 DOF	3 DOF 23	2 DOF 23
P_r /_P_ex_	20.4/4.8	19.7/5.5	19.8/5.3	18.9/6.1	12.1/3.0	11.6/3.4	11.8/3.1	11.4/3.5
PRI/PEI	14.2/1.1	14.0/1.3	14.1/1.2	13.8/1.6	9.2/0.5	9.0/0.6	9.1/0.6	8.9/0.7
U_r_/U_ex_	0.4/0.6	0.3/0.5	0.3/0.6	0.3/0.6	0.3/0.4	0.3/0.4	0.4/0.5	0.3/0.5
$P_{\infty }$	77	63	73	78	17	0	45	29
c	6.7	7.9	7.1	7.6	5.4	5.7	5.6	5.8
FCW	1.1	1.2	1.2	1.2	0.5	0.6	0.5	0.6
BCW	0.2	0.4	0.2	0.3	0.1	0.1	0.1	0.1
FEW	0.2	0.3	0.3	0.3	0.1	0.1	0.1	0.1

P_r_/P_ex_ indicate $P_{r}\max$ and $P_{\mathrm{ex}}\max$, respectively $\left[10^{3}\mathrm{Pa}\right]$; U_r_/U_ex_ indicate $U_{r}\max$ and $U_{\mathrm{ex}}\max$, respectively $\left[m\;\cdot \;s^{-1}\right]$; c $\left[m\;\cdot \;s^{-1}\right]$; $P_{\infty }$ [mmHg]; PRI/PEI $\left[10^{3}\mathrm{Pa}\;\cdot \;s\right]$; FCW, BCW, FEW refer to the corresponding areas $\left[J\;\cdot \;m^{-2}\right]$. Cases 1 and 2 have been selected to represent extreme cases based on $P_{r}\max$ values. F: Female.

In this study, $P_{r}$ and $P_{\mathrm{ex}}$ were calculated following,^5^ using only the pressure waveform measured at the carotid artery. We acknowledge that this approach presents a departure from the original reservoir-wave approach^2^; that is, without the contribution of the velocity waveform and with the measurement not taken at the aortic root. The validity of using only the pressure depends on two assumptions: (1) the diastolic pressure decay measured at different locations in the arterial system is very similar and (2) the $P_{\mathrm{ex}}$ is proportional to cardiac output at the aortic root. The aforementioned assumptions have been examined only in open chest dog experiments, and their clinical applicability is a matter of debate that can only be resolved through testing in humans. Regardless, in this work we followed the P-only approach as it is meant to make the calculations easier in the clinical setting, where the simultaneous measurement of P and U might be difficult.

Finally, whilst we acknowledge that the applicability of the reservoir-wave theory is still a matter of debate,^18,19^ it is important to consider that the scope of the study, the assessment of sensitivity of the derived parameters to choices made when fitting the reservoir pressure, is parametric in nature. As such this work is a quantitative contribution toward the ongoing discussion pertaining the determination of the asymptotic pressure, which requires fitting the diastolic pressure waveform or part of it.

### Limitations

In all of the reservoir-wave earlier work, the diastolic decay was fitted to a single exponential curve and we have recently studied the effect of changing the value of the single asymptotic pressure on the wave intensity parameters.^20^ However, fitting an exponential function can be perfomed via a multi-exponential approach, as noted in other areas of physical sciences. In fact, this possibility has been recently demonstrated using physiological data.^18^ Notewithstanding, the determination of the parameters pertaining the multi-exponential approach is more complex and cannot be established with a high degree of confidence.^21^ Therefore, in the current work we focused on the more common single exponential decay for the reservoir-wave approach.

Pressure and velocity measurements in this study were recorded sequentially and the two waveforms had to be aligned to satisfy conditions of the analysis. However, physiological changes either during or between the recordings were not implicated in this study and given that the time interval between recordings was short, it was safely considered that the hemodynamic parameters did not change significantly between recordings. Therefore, it can be carefully assumed that the sequential recordings of the data did not have negative effects on the analysis, results or conclusions.

## Conclusion

This quantitative study of hemodynamic and wave intensity parameters, under different fitting techniques for the reservoir pressure and velocity, demonstrated that the fitting method could bring significant variations in values and trends. Despite the changes in the shape of the $P_{r}$ waveform, its peak and time integral tended to remain constant in this study. This result is particularly relevant because both $P_{r}\max$ and PRI can be used in the clinical settings for calculating diagnostic indicators, such as the aortic augmentation index. The reservoir and excess velocity peaks, instead, changed more significantly. This outcome, together with the concomitant substantial change in excess pressure peak and integral, greatly affected wave intensity parameters. On the contrary, wave speed did not substantially change and seemed to be insensitive to the fitting techniques.

This study showed that reservoir pressure features and wave speed, being less substantially dependent on the fitting technique, could be more reliable diagnostic indicators.
